# A retrospective comparative evaluation of rectal preparation strategies for patients undergoing stereotactic body radiotherapy for prostate cancer

**DOI:** 10.1093/bjr/tqaf314

**Published:** 2025-12-22

**Authors:** Muoi N Tran, Giulio Didiodato, Amanda Lamb, Patrick Quinn, Janice Kim, Jessica Conway, Christiaan Stevens, Frederick Yoon, Jesse McLean, Adam Gladwish

**Affiliations:** Royal Victoria Regional Health Centre, Barrie, ON L4M 6M2, Canada; Royal Victoria Regional Health Centre, Barrie, ON L4M 6M2, Canada; Royal Victoria Regional Health Centre, Barrie, ON L4M 6M2, Canada; Royal Victoria Regional Health Centre, Barrie, ON L4M 6M2, Canada; Royal Victoria Regional Health Centre, Barrie, ON L4M 6M2, Canada; Royal Victoria Regional Health Centre, Barrie, ON L4M 6M2, Canada; Department of Radiation Oncology, University of Toronto, Toronto, ON M5S 1A1, Canada; Royal Victoria Regional Health Centre, Barrie, ON L4M 6M2, Canada; Department of Radiation Oncology, University of Toronto, Toronto, ON M5S 1A1, Canada; Royal Victoria Regional Health Centre, Barrie, ON L4M 6M2, Canada; Department of Radiation Oncology, University of Toronto, Toronto, ON M5S 1A1, Canada; Royal Victoria Regional Health Centre, Barrie, ON L4M 6M2, Canada; Royal Victoria Regional Health Centre, Barrie, ON L4M 6M2, Canada; Department of Radiation Oncology, University of Toronto, Toronto, ON M5S 1A1, Canada

**Keywords:** prostate stereotactic body radiation therapy, rectal preparation, polyethylene glycol 3350, fleet enema

## Abstract

**Objectives:**

We performed a retrospective study comparing 2 rectal preparation regimens, Polyethylene Glycol 3350 (PEG) and Fleet Enema (FE), in patients undergoing prostate stereotactic body radiation therapy (SBRT).

**Methods:**

The study included 24 patients receiving prostate SBRT (40 Gy in 5 fractions), for a total of 120 treatment fractions. Patients received either FE (*N = *73) or PEG (*N = *47) for rectal preparation. Outcomes included: (1) treatment time, measured from the initial setup cone-beam CT (CBCT) to the post-treatment CBCT (including rectal-related interventions, excluding machine delays); (2) intra-fraction motion, defined as the displacement vector between verification and post-treatment CBCTs registered to fiducial markers; and (3) clinical acceptability, determined by blinded review of all setup CBCTs by 3 radiation therapists (RTs), who scored each scan as either “Acceptable” (proceed directly to treatment) or “Need Intervention.” Regression analysis was used to compare regimens.

**Results:**

Population-averaged median treatment times were 14 minutes (95% CI, 5.8-22.2) for PEG and 11 minutes (95% CI, 9.6-12.3) for FE, with greater time variability in PEG (*P < .*001). Intra-fraction motion did not differ significantly between regimens. All 3 RTs judged the setup CBCTs as clinically acceptable for treatment 47.7% of the time (95% CI, 31.6%-63.8%) for the PEG regimen and 74.4% of the time (95% CI, 61%-87.8%) for the FE regimen.

**Conclusions:**

Overall, the FE regimen showed greater consistency in all outcome measures. This suggests an operational advantage for using FE since it results in more consistent patient treatment times without negatively impacting treatment quality and precision.

**Advances in knowledge:**

Daily FE improves the consistency of prostate SBRT treatment and enhances the clinical workflow by minimizing unplanned disruptions.

## Introduction

Prostate cancer is one of the most prevalent malignancies affecting men worldwide, with increasing demand for effective treatment options. Stereotactic body radiation therapy (SBRT) has emerged as a treatment option for men with low and intermediate risk prostate cancer.^1–3^ Given the increased dose per fraction and small planning target volume (PTV) margins, adequate dietary protocols and rectal preparation are critical to minimize intra-fraction prostate motion.^4–10^

Multiple studies have investigated strategies to reduce rectal volume variation. These include dietary protocols,^9^ daily enemas,^8^^,^^11–13^ a combination of antiflatulent diet and magnesia laxative,^14–17^ and gas-reducing probiotics.^18^ While maintaining rectal consistency is recognized as important, there is no clear consensus on which rectal preparatory regimen is most effective.^19^

Our institution implemented a prostate SBRT program in October 2020. At the outset, we employed the following strategy for patient rectal preparation: a fleet enema (FE) prior to CT simulation and fraction 1 of treatment, and subsequently 17 g of polyethylene glycol 3350 (PEG) daily throughout the rest of the treatment course. This approach was based on protocols from neighbouring institutions and prior literature suggesting limited dosimetric impact from strict rectal preparation.^20^ However, in practice we observed frequent difficulties in image matching associated with rectal distension (>4 cm). Consequently, after 6 months of experience (19 patients), the rectal preparation protocol was revised to daily FE prior to CT simulation and each treatment fraction.

We conducted a retrospective study using existing clinical data to systematically evaluate the 2 rectal preparation approaches. This study included patients treated with prostate SBRT between October 2020 and November 2021, excluding those used in the initial QA analysis. Our primary hypothesis was that daily pre-treatment FE preparation would result in a higher rate of favourable rectal anatomy for treatment and shorter treatment times compared with PEG. A secondary hypothesis was that FE preparation would also reduce intrafraction prostate motion compared to PEG.

## Methods

### Study population

Men with favourable intermediate-risk prostate cancer were treated with prostate SBRT at our institution if they met the following criteria: age >18 years, a pathologic diagnosis of prostate cancer, clinical stage ≤T2b, a biopsy showing ISUP Grade Group 1-2, ≤50% positive biopsy cores, and PSA levels ≤10.0 ng/mL. Additionally, prostate volume had to be ≤100 cc as measured by transrectal ultrasound at the time of biopsy. Patients also needed to tolerate both the transrectal ultrasound and the transperineal insertion of 4 gold fiducial markers.

For this retrospective analysis, we focused on patients treated between October 2020 and November 2021. From this population, a total of 24 patients were randomly selected, 12 treated during the first 6 months of the prostate SBRT program (prior to revising the rectal preparation strategy) and 12 treated after the revision. Each patient underwent 5 treatment fractions, resulting in 120 treatment fractions included in the study. The study was approved by our institutional Research Ethics Board (#R22-011).

### Radiation treatment planning

All patients received an educational pamphlet and were reminded to perform FE 2 days before the CT simulation (Philips Healthcare System, Cleveland, OH, United States) and the evening before the first treatment fraction. One hour prior to CT simulation, patients were instructed to drink 250 mL of water. As per institutional policy, fiducial marker insertion was performed on the same day, just before the CT simulation (2 mm slice thickness). Additionally, T1- and T2-weighted magnetic resonance (MR) images (Siemens Healthcare, Erlangen, Germany) were acquired following CT-simulation to aid in the target delineation.

Radiation treatment planning was performed using the Eclipse Treatment Planning System (Varian Medical Systems, United States). A 5-mm isotropic planning target volume (PTV) margin was applied to the clinical target volume (CTV), except for a 4-mm margin posteriorly. The CTV included the prostate and the base of the seminal vesicles. A prescription dose of 40 Gy in 5 fractions was delivered every other day. Fiducial markers were contoured with a 3-mm isotropic expansion to aid image guidance. All treatment plans utilized the volumetric modulated arc therapy (VMAT) technique, delivered with 2 coplanar arcs. All 24 patients were planned and treated in the same manner.

### Radiation treatment

**Figure 1.**
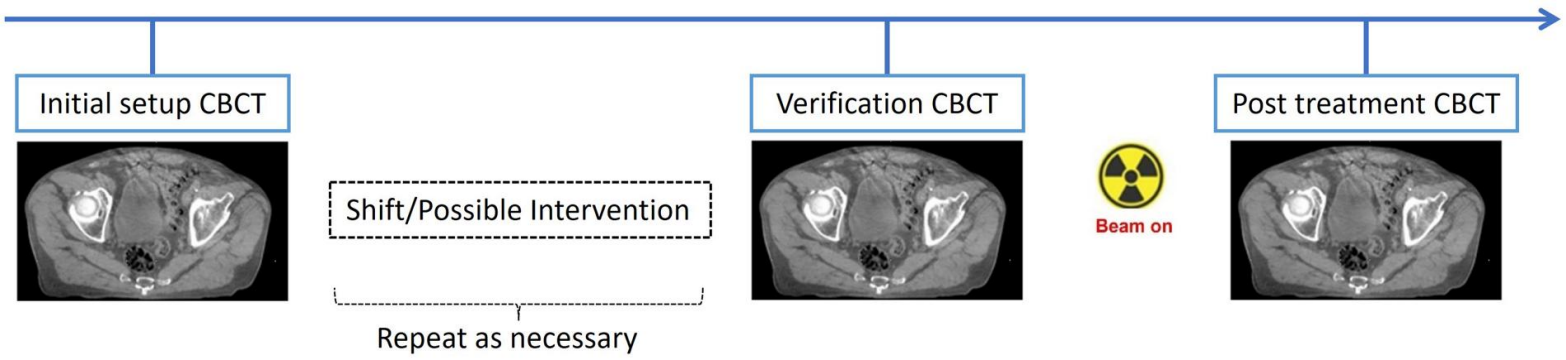
Workflow for CBCT-guided treatment for prostate SBRT. Abbreviations: CBCT = cone-beam CT; SBRT = stereotactic body radiation therapy.

Patients were treated using a TrueBeam linear accelerator (LINAC) equipped with a 6-degree-of-freedom couch (Varian Medical Systems, United States). According to our institutional protocol, initial patient setup was performed using skin tattoos for alignment, followed by cone-beam computed tomography (CBCT). Alignments followed these priorities: (1) registering fiducial markers within their margins, (2) assessing the prostate-rectal interface, and (3) ensuring the prostate was fully contained within the PTV. A verification CBCT was acquired before beam-on, with an additional CBCT performed after each treatment fraction. If the initial CBCT revealed rectal gas or stool that interfered with target visualization or alignment, ad hoc interventions were performed (eg, asking the patient to void or walk to relieve gas, or inserting a rectal tube), followed by a repeat CBCT. Figure 1 illustrates the treatment process, from the initial setup CBCT to completion of each fraction.

### Outcome metrics

The treatment fractions were categorized into the PEG regimen or the FE regimen. The outcomes are determined as follows:

1. Treatment time
  The total treatment times for each treatment fraction were extracted from the time stamps of CBCT image acquisition, measured from the start of the initial setup CBCT to the start of the post-treatment CBCT. This included the time required for rectal related interventions but excluded any breaks unrelated to patient factors (eg, machine breakdowns).
2. Intra-fraction motion
  The verification CBCT was registered with the post-treatment CBCT using Varian’s Image Registration Point Match module (Varian Medical Systems, United States), with fiducial markers serving as the match points. Deviations in all 3 directions (Δ*x*, Δ*y*, Δ*z*) were recorded. Since the images were acquired before and after beam delivery (Figure 1), with no couch movement in between, the differences between the 2 CBCTs represented pure intra-fraction motion. This motion could result from patient movement and/or physiological changes (eg, gas).
  The intra-fraction motion was characterized in 3 dimensions by the magnitude of the displacement vector, calculated as
  $$\mathrm{Displacement}=\;\sqrt{\Delta x^{2}+{\Delta y}^{2}+{\Delta z}^{2}\;}.$$
3. Acceptability score

To eliminate observer bias and minimize the influence of confounding clinical factors at the time of treatment—such as radiation therapists (RTs)’ experience, scheduling constraints, and other operational variables—we conducted a controlled retrospective treatment experiment. Three expert RTs, blinded to the rectal preparation regimen, independently reviewed a total of 120 setup CBCTs. Each scan was categorized as either “Acceptable” or “Need Intervention.”

An “Acceptable” designation would indicate that the RTs approved the match, meaning that the alignment priorities (fiducial markers, prostate-rectal interface, and prostate coverage within the PTV) were satisfied, and would proceed directly to verification CBCT and treatment (Figure 1). In contrast, a “Need Intervention” score signified that additional measures, as outlined in the Radiation Treatment section, were required. This approach ensured a standardized evaluation process independent of clinical or operational pressures and the treating RT’s knowledge of which rectal preparation was being used at the time of treatment.

### Statistical analysis

Sample statistics for all continuous variables were described using medians and interquartile ranges given the skewed data distribution. Quantile regression analyses for the median (50th quantile) was used to model outcome variation given the skewed distribution along with the presence of significant outliers. The independent variable of interest was the rectal preparation regimen, FE versus PEG. Post-regression estimates of population-averaged effects were reported for all continuous outcome variable.

To assess the consistency of the Acceptability score determination by the 3 RTs, the inter-rater agreement was measured using kappa statistics. Pairwise agreement was assessed between RT1-RT2, RT2-RT3, and RT3-RT1. Complete agreement is defined as all 3 RTs categorize the initial setup CBCT in the same way, designating it as either “Acceptable” or “Need Intervention.” Adjusted differences in proportions of subjects treated with different rectal preparations in whom there was variable agreement between RT Acceptability were estimated using ordered logistic regression analysis. Robust standard errors were used to account for possible clustering of measurements between subjects.

## Results

There were 24 subjects constituting 120 treatment fractions in the final sample, with no missing data. Twelve subjects had 5 FE regimens in all 5 fractions, 11 subjects had 1 FE regimen followed by 4 PEG regimens, and 1 subject had FE for fractions 1 and 5, and PEG for fractions 2 to 4. Overall, there were 47 PEG regimens and 73 FE regimens.

**Figure 2.**
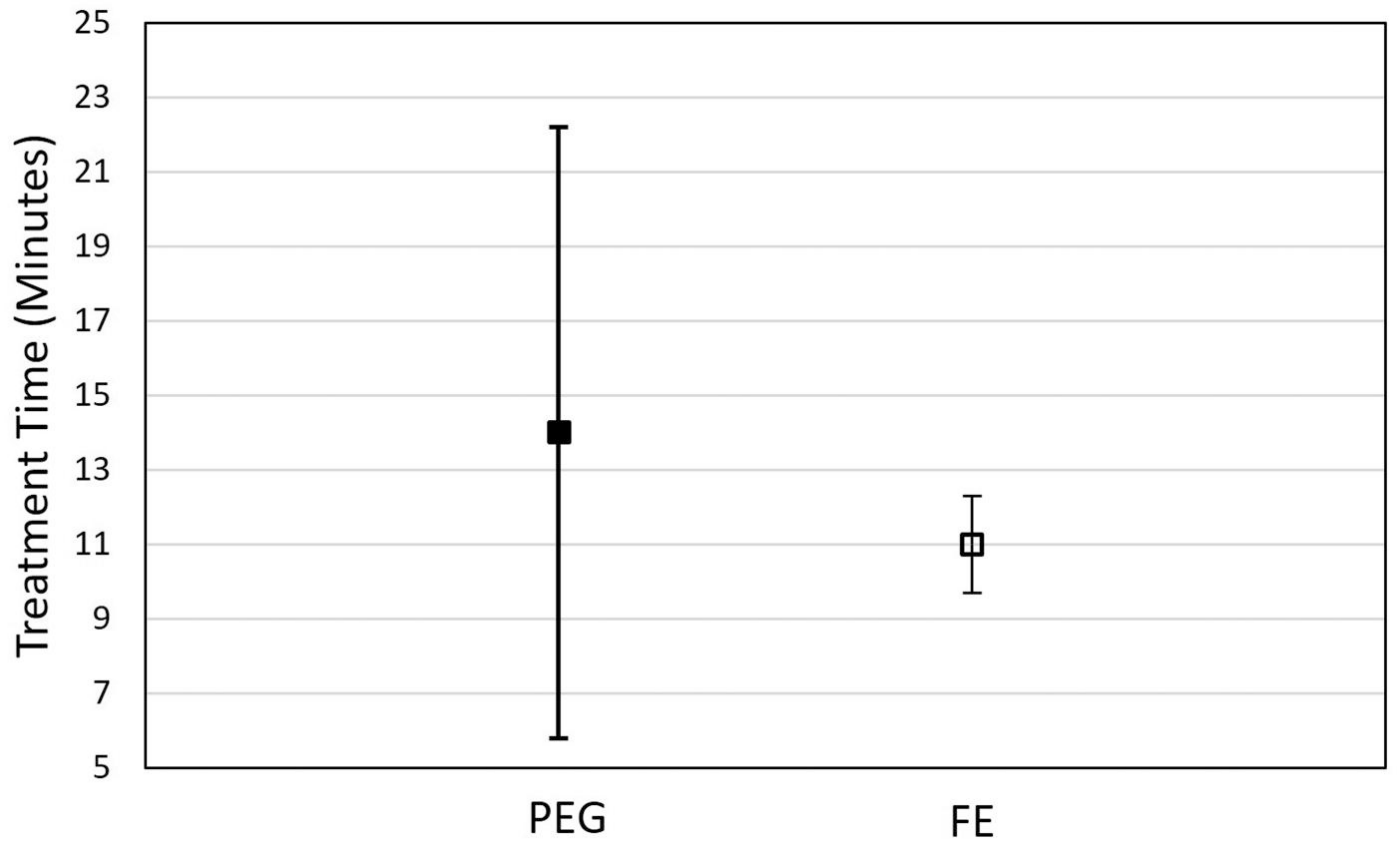
Population-averaged median treatment times by rectal preparation: 14 minutes (95% CI, 5.8-22.2) for the PEG regimen versus 11 minutes (95% CI, 9.6-12.3) for the FE regimen. Abbreviations: FE = Fleet Enema; PEG = Polyethylene Glycol 3350.

**Figure 3.**
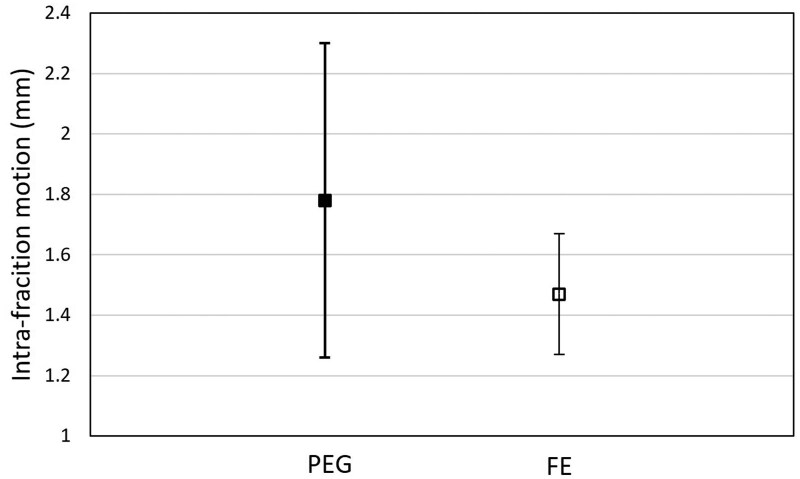
The population-averaged intra-fraction motion was 1.78 mm (95% CI, 1.26-2.30) for the PEG regimen and 1.47 mm (95% CI, 1.27-1.67) for the FE regimen. Abbreviations: FE = Fleet Enema; PEG = Polyethylene Glycol 3350.

Both treatment time and intra-fraction motion data were right-skewed. Based on the regression modelling, the population-averaged median treatment times were 14 minutes (95% CI, 5.8-22.2) and 11 minutes (95% CI, 9.6-12.3) for the PEG and FE regimen, respectively (Figure 2). Although the difference between the population-averaged treatment times was not statistically significant, the treatment time variance was significantly lower in the FE regimen (*P < .*001), suggesting a possible operational advantage for using FE due to less variability. The population-averaged intra-fraction motion was 1.78 mm (95% CI, 1.26-2.3) and 1.47 mm (95% CI, 1.27-1.67) for the PEG and FE regimen, respectively (Figure 3). There was no statistically significant difference between rectal preparation in the population-averaged intra-fraction motion regimens.

Among the RTs, there was complete agreement on the Acceptability scores in 91 out of 120 cases, with 76 in the “Acceptable” score and 15 in the “Need Intervention” score. The agreement rates among RT pairs ranged from 81% to 88%, with an overall kappa statistic of 0.5638 (*P < .*001) indicating moderate agreement.

**Figure 4.**
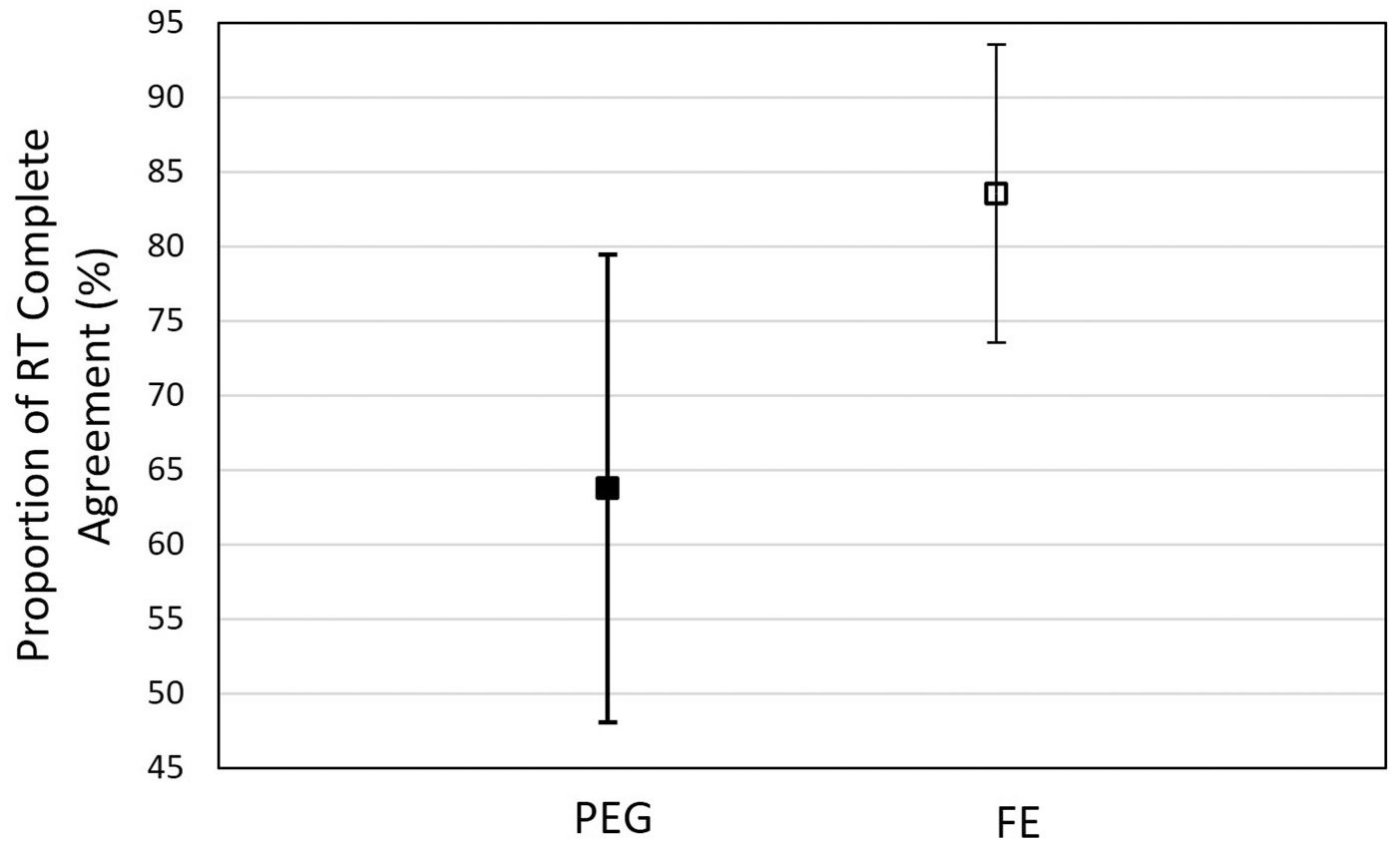
RTs are in complete agreement 63.8% (95% CI, 48.2-79.4) of the time in the PEG regimen and 83.6% (95% CI, 73.5-93.6) of the time in the FE regimen. Abbreviations: FE = Fleet Enema; PEG = Polyethylene Glycol 3350; RT = radiation therapist.

**Figure 5.**
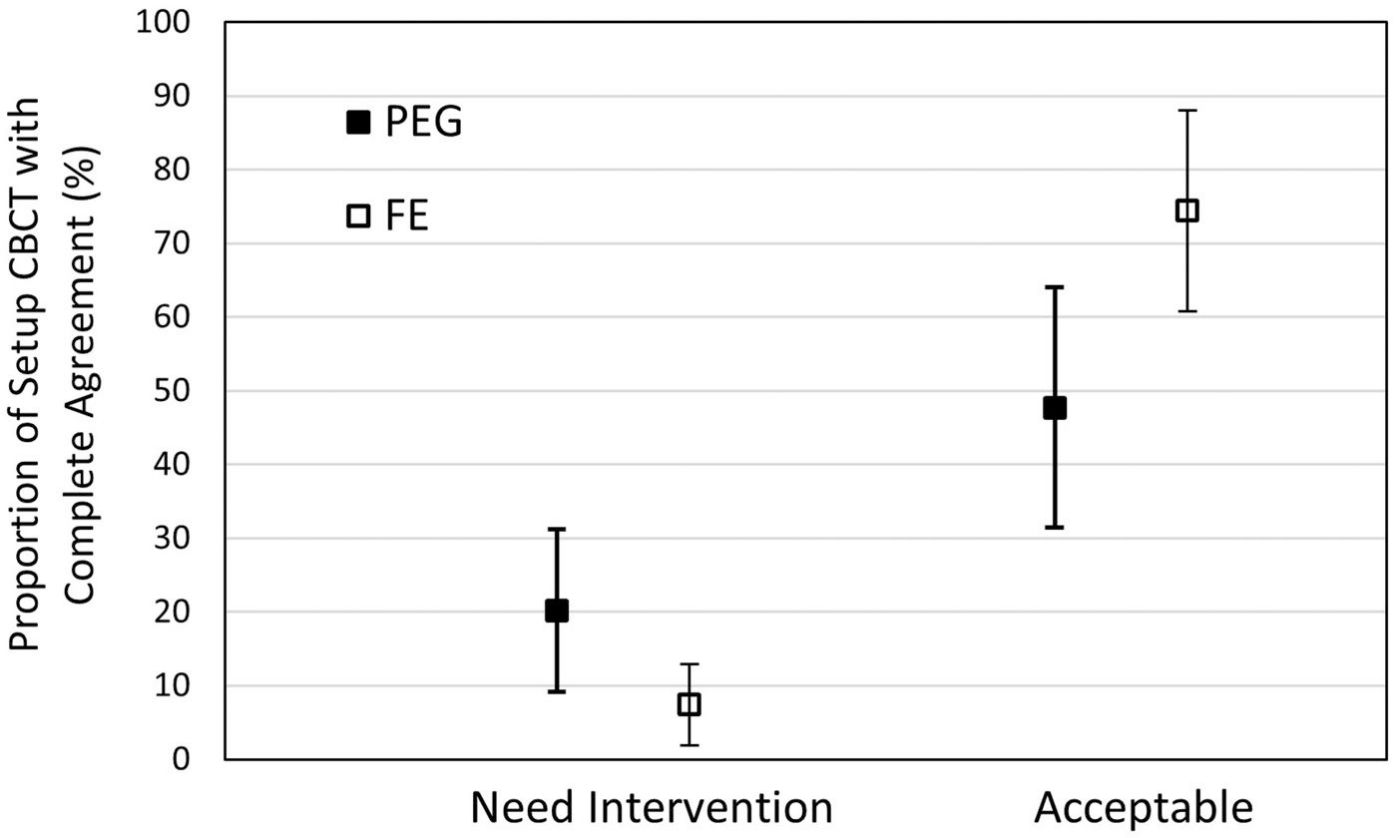
Probability of setup CBCT with complete agreement by rectal preparation: In the PEG regimen, 20.2% (95% CI, 9.2-31.2) of the time all 3 RTs scored the initial setup CBCTs as “Need Intervention” and 47.7% (95% CI, 31.4-64) of the time as “Acceptable.” In contrast, 7.4% (95% CI, 1.9-12.9) of the time they scored the setup CBCTs as “Need Intervention” and 74.4% (95% CI, 60.8-88) of the time as “Acceptable” in the FE regimen. Abbreviations: CBCT = cone-beam CT; FE = Fleet Enema; PEG = Polyethylene Glycol 3350; RT = radiation therapist.

The probability of RT in complete agreement on the Acceptability score differed between rectal preparations, with the FE group showing a 19.73% (95% CI, 2.43-37.03) higher probability of agreement compared to PEG (Figure 4). When considering only cases with complete agreement (*N = *91), the proportion of setup CBCTs scored as “Acceptable” was higher than those scored as “Need Intervention” for both regimens; however, CBCTs were 26.7% more likely to be rated “Acceptable” in the FE group than in the PEG group (Figure 5). There were no significant differences between regimens among cases with disagreement.

## Discussion

Implementing daily FE reduced treatment time variability and improved workflow predictability compared with PEG. Although the difference in the treatment duration was not statistically significant, the variance was notably reduced within the FE cohort. In an era where clinics often face staffing shortages, predictable treatment durations are essential for smoother workflows and optimized resource utilization.

It is important to clarify that the regression-derived estimates reflect population-averaged treatment times, and not the raw treatment times. In the raw dataset, the shortest observed treatment time (7.5 minutes) occurred in a PEG fraction; however, the same patient also had other PEG fractions lasting 9.5, 29.5, and 41 minutes. Similarly, the patient in the PEG group with the longest observed treatment (>90 minutes) also had PEG fractions lasting 8, 31.5, and 39 minutes. Extremes in treatment duration occurred in both regimens but were less frequent with FE. Patient voiding during treatments occurred in both groups but was less common with FE (15% of FE fractions vs. 32% of PEG fractions). When voiding occurred, it necessitated patient repositioning and repeated CBCTs, contributing substantially to prolonged treatment sessions. Other factors prolonging treatment times included time to reach a consensus between treatment therapists and additional CBCTs after the initial verification. Taken together, these observations suggest that while short treatment times occasionally occurred in the PEG group, they were less consistent and more likely due to chance. This supports our hypothesis that FE confers improved predictability and workflow consistency, even if the absolute shortest treatment times may still occasionally occur with PEG.

We note that most prior studies of rectal preparation report effects on rectal volume, rectal gas, and prostate intra-/inter-fraction motion, rather than per-fraction treatment duration.^8–19^ CyberKnife and some online-adaptive robotic workflows are reported to have substantially longer per-fraction durations (typically 40-60 minutes)^21^^,^^22^ which can increase the opportunity for intrafraction motion, but these reports do not evaluate bowel-preparation effects on fraction duration. To our knowledge, no peer-reviewed study directly compares per-fraction treatment time between sessions with and without bowel preparation for prostate SBRT—therefore, our analysis of treatment-time variability with FE addresses a gap in the literature.

Our analysis of intra-fraction data revealed a maximum displacement of 2.3 mm in the PEG group, and 1.67 mm in the FE group, consistent with our expectations, given that our image-matching protocol requires fiducial markers to remain within a margin of 3 mm. These values fall within the range reported across delivery systems. For example, McNeice et al,^23^ reported an average root mean square intra-fraction motion of 2.5 mm in prostate SBRT patients treated with daily CBCT. Xie et al^24^ reported mean intra-fraction motion of ∼2 mm in a CyberKnife cohort. It should be noted that CyberKnife studies involve continuous tracking over longer delivery times, which allows more detailed motion characterization compared with conventional linac CBCT; thus, direct comparisons with our workflow should be interpreted with this context in mind.

Although the mean displacement did not differ significantly between cohorts, the variance was reduced in the FE group, a trend also observed in the treatment time data. A study by Nichol et al,^14^ using cine-MRI in 42 patients under a milk-of-magnesia plus antiflatulent diet regimen, found that the prostate was displaced >3 mm for ∼11%-12% of the time in both baseline and treatment with prep scans, indicating that the regimen did not significantly reduce intra-fraction motion. In contrast, Choi et al^13^ reported that daily enemas reduced prostate motion substantially, with a mean intra-fraction motion of 1.11 ± 0.77 mm, with 97.6% of fractions showing ≤3 mm displacement. Our FE cohort demonstrated a mean displacement of 1.47 mm with lower variance and a maximum displacement 1.67 mm, compared with 2.3 mm in the PEG group. While our mean value is somewhat higher than Choi et al,^13^ the key observation is the marked reduction in variance relative to PEG, suggesting that FE provides more reproducible motion control. Taken together, these findings suggest that FE confers motion stability and reproducibility benefits, while also improving efficiency and workflow predictability in daily clinical practice.

The results from the controlled experiment were particularly noteworthy. RTs were in complete agreement 20% more frequently for treatment fractions in which patients received FE. Moreover, when they were in complete agreement, the setup CBCTs in the FE regimen were 26.7% more likely to be “Acceptable” than those in the PEG regimen. This finding aligns with our observations of treatment times, suggesting a reduction in overall treatment duration. This reduction directly translates to a significant enhancement in clinic efficiency, enabling more predictable workflows and optimized resource utilization.

There are a couple of limitations in our assessment of intra-fraction motion. First, we used the fiducial marker displacement as a surrogate for prostate motion, assuming marker stability between the planning CT scan and the treatments, which are typically 2 weeks apart at our institution. Second, intra-fraction motion consists of both rigid and deformable components, but our measurements primarily captured rigid prostate motion, characterized by the displacement of the 3-4 implanted fiducial markers. Xie et al^24^ found prostate deformation to be of minimal concern based on their investigation of the Rigid Body Error profiles of the fiducial markers, supporting the validity of our results. Furthermore, these limitations apply equally to both the FE and PEG cohorts, so they are unlikely to alter the findings of this study. Finally, we did not directly quantify patient experience. While we assume that reduced treatment times and greater consistency contribute to an improved patient experience, this remains to be explicitly evaluated in future research.

## Conclusions

Our study demonstrates that the daily administration of FE significantly improves the consistency of treatment times and streamlines clinical workflows. The use of FE yielded better consensus among treatment staff when evaluating CBCT setup images and was more likely to be deemed acceptable for treatment, when compared to PEG. Integrating daily FE into our simulation and treatment protocols minimized treatment disruptions and reduced variability, contributing to enhanced precision in radiation delivery. These improvements not only optimize operational efficiency but may also support better treatment outcomes by ensuring more consistent and reliable care for patients. Our findings support consideration of daily FE as a standard rectal preparation regimen in prostate SBRT workflows.

## Data Availability

Research data are stored in an institutional repository and will be shared upon request to the corresponding author.
